# Research progress on heat stress response mechanisms in *Aspergillus niger*

**DOI:** 10.3389/fmicb.2026.1750016

**Published:** 2026-02-04

**Authors:** Yongtao Pan, Jun Li

**Affiliations:** College of Food Science and Technology, Hebei Normal University of Science and Technology, Qinhuangdao, China

**Keywords:** antioxidant defense, *Aspergillus niger*, compatible solutes, heat stress response, HSPs, metabolic reprogramming, thermotolerance

## Abstract

*Aspergillus niger*, an industrial filamentous fungus recognized as GRAS (Generally Recognized as Safe) and vital for food fermentation and enzyme production, has an optimal fermentation temperature around 30 °C; however, heat stress in industrial systems impairs its cellular viability and reduces target product synthesis efficiency. This review systematically summarizes the multi-level coordinated heat stress response mechanisms of *A. niger* by integrating existing research findings, revealing that the fungus copes with heat stress via cell membrane remodeling, rapid accumulation of compatible solutes, cAMP/PKA-mediated metabolic reprogramming, protein quality control, and activation of antioxidant defense systems. These mechanisms synergistically enhance *A. niger*’s heat resistance, while current research still lacks data on early stress signaling events, complete PKA downstream regulatory networks, and multi-omics integration. The review’s innovation lies in identifying potential adaptive strategies specific to eukaryotic filamentous fungi (e.g., non-classical membrane regulation) and providing a theoretical basis for improving *A. niger*’s thermotolerance through metabolic engineering.

## 1 Introduction

*Aspergillus niger* is an industrially important filamentous fungus, with numerous strains classified as GRAS (Generally Recognized as Safe) by the U. S. Food and Drug Administration (Schuster et al., 2002). *A. niger* features a robust enzymatic system that facilitates the efficient synthesis of various metabolites, ranging from organic acids like citric and gluconic acids (Laothanachareon et al., 2018; Behera, 2020) to diverse enzymes such as pectinases, amylases, and cellulases (Cairns et al., 2018; Bellaouchi et al., 2021). This capacity reinforces its pivotal role in food fermentation and the production of industrial additives, leading to its designation as a “cell factory” (Yu et al., 2021).

During the fermentation process, a substantial amount of thermal energy is released due to the growth and metabolism of microorganisms, leading to an elevation in the temperature of the fermentation broth (Zhang et al., 2023). The optimal growth temperature of *A. niger* is 37 °C (Szewczyk and Myszka, 1994), while for various industrial fungi including *A. niger*, the optimal temperature for their fermentative production of organic acids and enzymes is approximately 30 °C (Santos et al., 2016; Darabzadeh et al., 2019; Akhter et al., 2022; Nayab et al., 2022). If temperature control is inadequate during the late stages of microbial fermentation and metabolism, the temperature of the fermentation system may rise above 50 °C. As a critical environmental factor regulating the physiological metabolism of fungal cells, abnormal temperature elevation exerts multiple adverse effects on cells, with one of the most prominent consequences being a sharp decline in cellular viability (Bhabhra and Askew, 2005), thereby directly reducing the synthesis efficiency of target products. In industrial production, large quantities of cooling water are commonly used for temperature control. Coupled with the global warming trend, especially in summer, the high ambient temperature leads to a significant increase in cooling water consumption, which in turn raises industrial energy consumption.

Investigating the effects of heat stress on the cellular structure and physiological metabolism of *A. niger*, as well as elucidating its underlying response mechanisms, has significant implications for enhancing the thermotolerance of fermentation strains and reducing industrial energy consumption. Although an increasing number of studies have examined heat stress in filamentous fungi, the potential mechanisms underlying their responses have not been systematically detailed. This review aims to comprehensively summarize the research progress regarding the heat stress response mechanisms of *A. niger*, discuss existing research gaps and future directions, and provide a robust theoretical foundation for the targeted improvement of thermotolerance in industrial strains through metabolic engineering and synthetic biology strategies.

## 2 Remodeling of cell membrane components

The cell membrane is one of the first cellular structures to sense heat stress (Digel, 2011). High temperatures directly cause excessive increases in membrane fluidity, impair its selective permeability barrier function, trigger ion leakage, and induce denaturation and inactivation of membrane proteins, ultimately leading to cell damage or even death (Grundy et al., 2015). Therefore, *A. niger* must activate a series of complex adaptive mechanisms to remodel the cell membrane and maintain the stability of its structure and function.

### 2.1 Fatty acid unsaturation: a challenge to the classical theory

In the field of microbial temperature adaptation research, the concept of “Homeoviscous Adaptation” serves as a fundamental theoretical framework (Sinensky, 1974; Hazel, 1995). This model posits that cells regulate the degree of unsaturation of fatty acid chains in membrane lipids to maintain optimal membrane fluidity across a range of temperatures. When subjected to elevated temperatures, cells typically reduce the unsaturation level of fatty acids, resulting in an increased proportion of saturated fatty acids. This modification enhances intermolecular van der Waals forces, thereby decreasing membrane fluidity (Sinensky, 1974; Haddaji et al., 2015).

While this theory has been widely validated in bacteria and yeasts, its applicability is challenged in *A. niger*. Multiple studies analyzed the membrane lipids of *A. niger* after heat shock, and the results demonstrated that there was no significant increase in the content of unsaturated fatty acids, nor was there any obvious change in the proportion of saturated fatty acids in its major phospholipid components (Tereshina et al., 2010, 2011; Ianutsevich and Tereshina, 2019; Ianutsevich et al., 2020). However, these analyses were conducted at 40 ~ 41 °C, and whether the same pattern will still be exhibited under higher temperature stress requires further verification.

This phenomenon suggests that *A. niger* may not follow the classical membrane fluidity regulation pathway, but instead relies on alternative mechanisms to maintain cell membrane stability. This thus leads to the hypothesis that *A. niger* probably represents a unique “lipid-domain” adaptive strategy specific to eukaryotic filamentous fungi, which differs from the adaptive patterns of organisms such as bacteria and yeasts.

### 2.2 Stability of sterol content

Sterols are key components maintaining cell membrane structure and fluidity. Their flat, rigid cyclic structure can embed into the phospholipid bilayer, acting as “fluidity buffers” (Jordá et al., 2022). In contrast to the dynamic changes of phosphatidic acid (PA) and sphingolipids (SLs), the sterol content in the cell membrane of *A. niger* remains relatively stable under heat stress. Studies have shown no significant increase in sterol levels after heat stress treatment at 40 ~ 41 °C (Tereshina et al., 2010). Another experiment using radioactive labeling also indicated that the synthesis rate of cholesterol (as a representative of sterols) remains essentially unchanged during heat stress (Tereshina et al., 2013). These results suggest that the heat tolerance strategy of *A. niger* does not depend on increasing sterol content to enhance membrane rigidity.

### 2.3 Accumulation of PA and SLs

**Figure 1:**
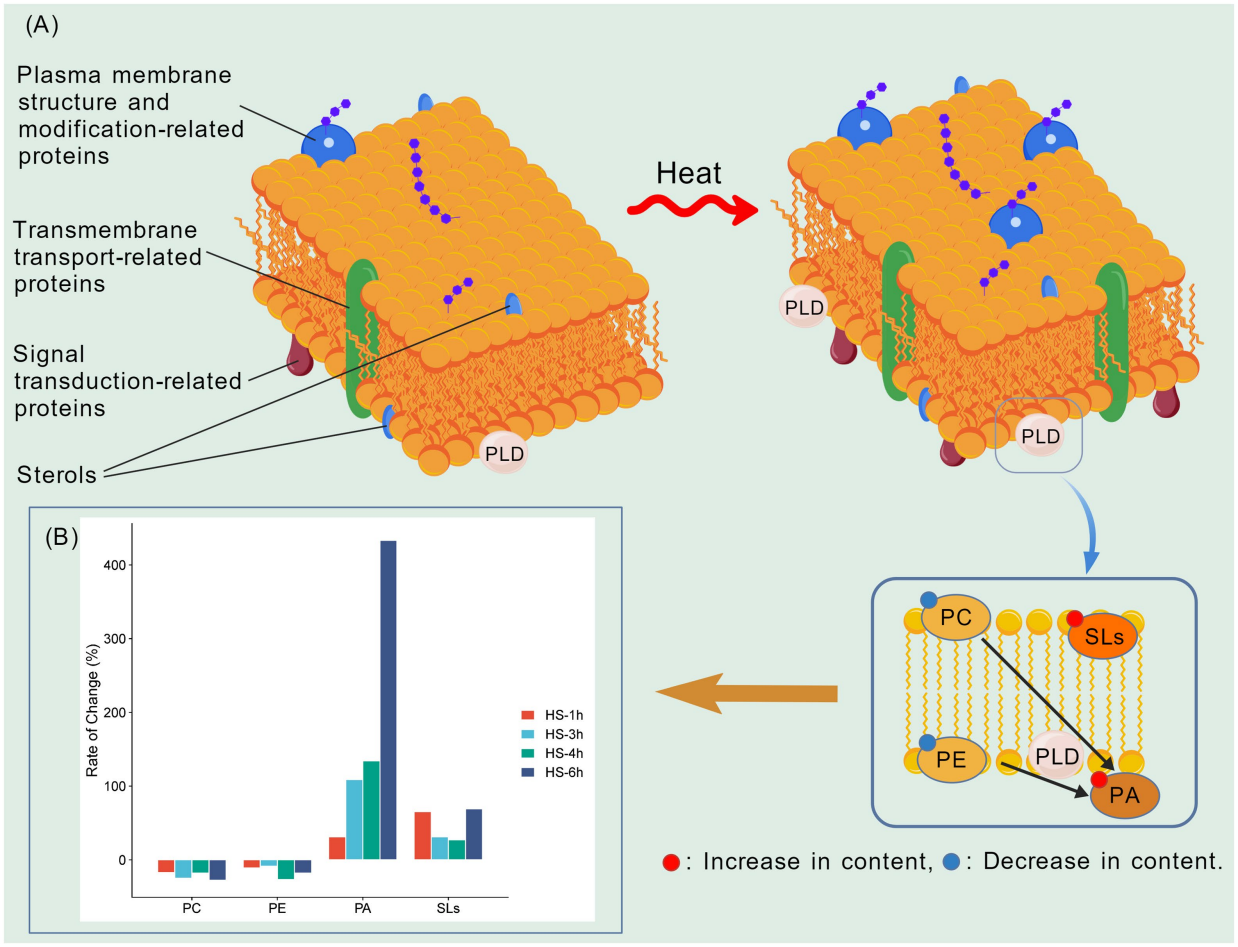
Heat-triggered lipid remodeling in *Aspergillus niger*. **(A)** Membrane lipid remodeling and dynamic changes in *A. niger* under heat stress. PC refers to phosphatidylcholines; PE denotes phosphatidylethanolamines; PA indicates phosphatidic acids; SLs represent sphingolipids; and PLD stands for phospholipase D. **(B)** Changes in membrane lipid components under heat stress at 40–41 °C at various time points. All percentages are directly derived from the quantitative results in Tereshina et al. (2010, 2011, 2013).

Multiple studies consistently demonstrate that a significant increase in the contents of PA and SLs is one of the core characteristics of *A. niger* in responding to heat stress. Experimental evidence shows that when *A. niger* mycelia undergo heat stress from the normal growth temperature to 40 ± 1 °C, the content of PA (Ianutsevich and Tereshina, 2019) and SLs (Tereshina et al., 2011) in their cell membrane components increase significantly. An in-depth study based on isotope labeling revealed that the labeled amount of PA increased by more than threefold under heat stress conditions (Tereshina et al., 2013), whereas the contents of major phospholipids [e.g., phosphatidylcholine (PC) and phosphatidylethanolamine (PE)] decreased significantly (Ianutsevich et al., 2020) (Figure 1B).

The accumulation of PA may result from the degradation of major phospholipids, indicating that phospholipase D (PLD) plays a critical role in PA biosynthesis. As a membrane-localized enzyme, PLD directly catalyzes the hydrolysis of PC and PE to produce PA (Morris, 2019). An increased expression level of PLD protein has been observed under heat stress (see Supplementary Table 1), suggesting that *A. niger* may utilize PA in signal transduction during the heat stress response. As a lipid second messenger, PA is extensively involved in various cellular processes, including cytoskeletal rearrangement, vesicular transport, cell cycle progression, apoptosis, and cell wall remodeling (Liu et al., 2013; Thakur et al., 2019). Moreover, PA interacts with a variety of proteins, such as protein kinases and small GTPase regulators (Liu et al., 2013), potentially transducing heat signals downstream by modulating the enzymatic activity of target proteins or enhancing their localization to membranes. Furthermore, the conical structure of PA enables it to induce negative membrane curvature, facilitating membrane fusion and fission (McMahon and Gallop, 2005; Tanguy et al., 2019), which are crucial for plasma membrane remodeling under heat stress.

Similar to PA, the total content of SLs also shows an increasing trend under heat stress (Tereshina et al., 2010, 2011, 2013) (Figure 1B). This phenomenon is highly conserved with the well-established heat stress response mechanism in *Saccharomyces cerevisiae* (Dickson et al., 1997; Jenkins et al., 1997; Chen et al., 2013). Notably, SLs, especially glycosphingolipids, can assemble into lipid raft microdomains through hydrogen bond interactions with sterols (Jiang et al., 2021). These microdomains possess higher structural order and rigidity, which in turn enhances the stability of the entire cell membrane under elevated temperature conditions.

### 2.4 Response of membrane-associated proteome

Studies on *A. niger* using proteomic techniques have revealed extensive changes in the protein expression profile under heat stress. It was found that a large number of differentially expressed proteins (DEPs) were identified under heat stress at 50 °C (Deng et al., 2020). Further screening showed that a total of 57 DEPs are plasma membrane component proteins, which can be classified into transmembrane transport-related proteins, signal transduction-related proteins, and plasma membrane structure and modification-related proteins based on their functions (Figure 1A). These include small G proteins (e.g., RAS GTPase), signal transduction proteins (e.g., G protein α-subunit), and various transporters (e.g., ABC transporters) (Supplementary Table 1). In particular, the upregulation of RAS GTPase and G protein subunits suggests that G protein-mediated signaling pathways may play an important role in heat stress perception.

However, the existing proteomic data are only based on stress treatment at a relatively long time point (24 h), lacking detailed characterization of dynamic changes in the early stage of heat stress (0–30 min). Identification of membrane-associated proteins (e.g., transporters, signal receptors, lipid synthases, etc.) with altered abundances within these critical time windows will provide important molecular evidence for understanding the immediate and adaptive processes of heat stress response.

## 3 Accumulation of compatible solutes

During biological evolution, compatible solutes have been preserved by natural selection owing to their distinctive physicochemical properties (Jain and Roy, 2009). These small molecules can sustain cellular osmotic balance through osmotic adjustment and enhance cellular tolerance to environmental stresses by stabilizing the native conformations of biomacromolecules, including proteins and lipid membranes, without disrupting normal physiological and metabolic processes (Yancey, 2005). In *A. niger*, trehalose and mannitol, as two principal types of compatible solutes, confer exceptional stress resistance to conidia through a synergistic effect.

### 3.1 Positive correlation between trehalose accumulation and heat tolerance

As a non-reducing disaccharide, trehalose constitutes approximately 3.6% of the dry weight of *A. niger* conidia (Witteveen and Visser, 1995). It functions not only as an energy reserve that participates in metabolic regulation (Shi et al., 2010) but also enhances cellular tolerance to extreme environments by stabilizing the native conformations of biomacromolecules, including proteins and lipid membranes. This stabilization is particularly evident under conditions of heat stress (Li et al., 2009), oxidative stress (Kieliszek et al., 2020), salt stress (Mahmud et al., 2009), desiccation stress (Tapia et al., 2015), and various other environmental challenges. Trehalose can prevent dehydration by substituting for the “hydration shell” surrounding macromolecules (Ajito et al., 2018) and can inhibit the aggregation of protein molecules denatured by heat stress (Singer and Lindquist, 1998). It reduces protein aggregation and maintains polypeptide chains in a partially folded state, thereby facilitating their refolding by cellular chaperones (Benaroudj et al., 2001).

**Figure 2:**
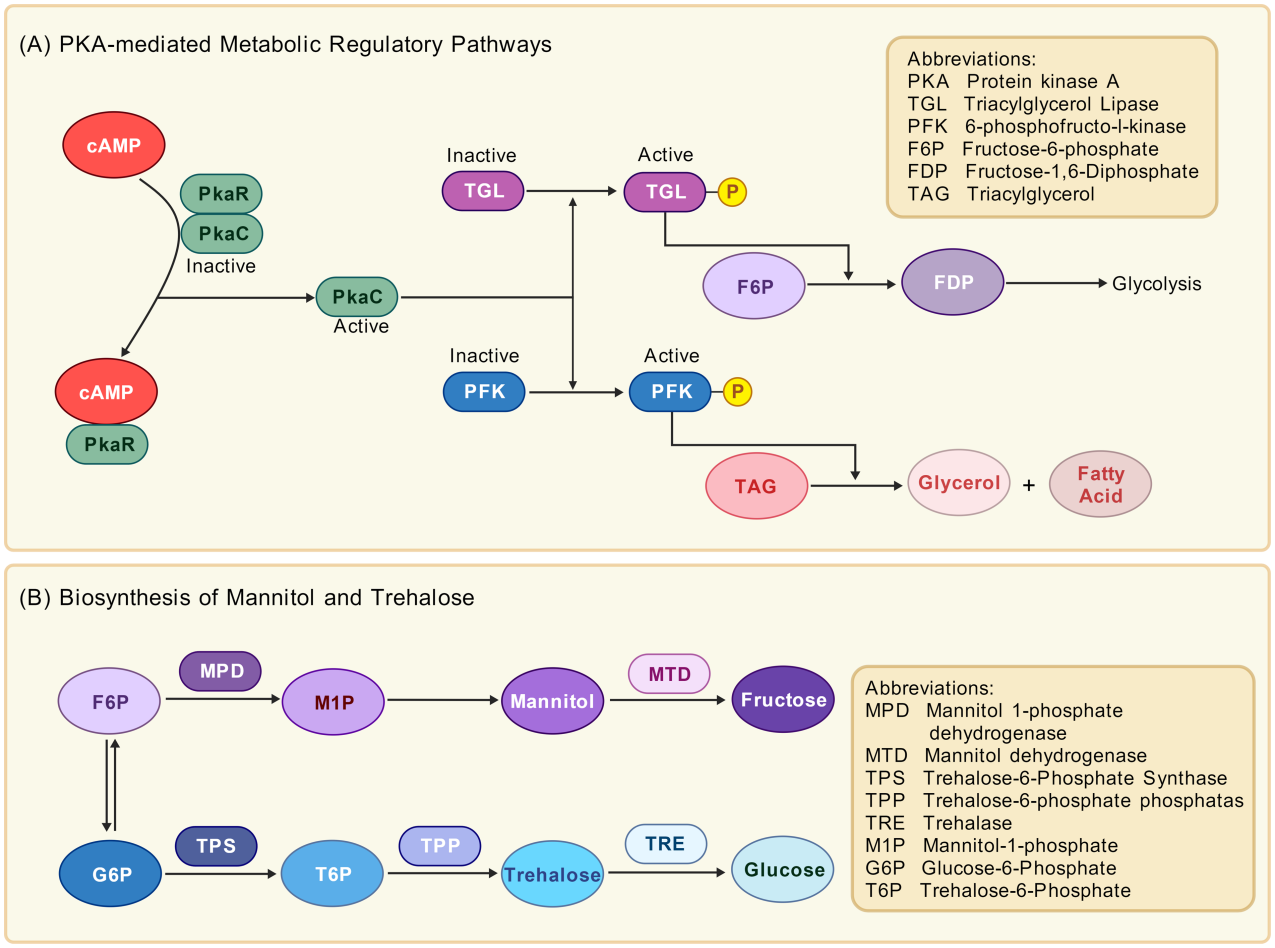
Schematic diagrams of metabolic regulatory pathways in *Aspergillus niger*. **(A)** The PKA-mediated metabolic regulatory pathway demonstrates the cAMP-dependent activation of PKA, which regulates triacylglycerol lipase and 6-phosphofructokinase through phosphorylation. This pathway underscores the roles of triacylglycerol lipase and 6-phosphofructokinase in glycolysis and triacylglycerol metabolism. **(B)** The biosynthetic pathways for mannitol and trehalose outline the critical enzymatic reactions involved in synthesizing mannitol and trehalose from fructose-6-phosphate and glucose-6-phosphate, respectively.

In fungi, the biosynthetic pathway of trehalose follows the classical trehalose-6-phosphate synthase/phosphatase dual-enzyme system (Van Ende et al., 2021). The specific synthesis steps are as follows: first, trehalose-6-phosphate synthase (TPS) catalyzes the reaction between glucose-6-phosphate (G6P) and uridine diphosphate glucose (UDPG) to generate trehalose-6-phosphate (T6P); subsequently, trehalose-6-phosphate phosphatase (TPP) dephosphorylates T6P to produce trehalose (Figure 2B).

Multiple studies have clearly demonstrated that when *A. niger* is exposed to heat stress below the lethal temperature threshold for a duration shorter than the temperature-specific lethal time threshold, its cells will rapidly accumulate trehalose (Tereshina et al., 2010, 2011; Svanström and Melin, 2013; Ianutsevich and Tereshina, 2019; Ianutsevich et al., 2020), and this accumulation underpins its acquired thermotolerance. Reverse genetic evidence further reinforces the central role of trehalose in heat protection. It has been found that trehalose biosynthesis-deficient *A. niger* mutants constructed via approaches such as gene knockout exhibit significantly lower survival rates under heat stress conditions compared to the wild-type strain. For instance, the intracellular trehalose content of *tpsA* gene deletion mutants is approximately 44% of that in the wild type, and these mutants show higher sensitivity to heat stress (Wolschek and Kubicek, 1997). Similarly, *tppB* gene deletion mutants also display increased sensitivity to heat stress due to reduced trehalose levels (Svanström et al., 2014) (Table 1).

**Table 1:** Intracellular trehalose content and survival rate of key gene mutants in various trehalose metabolic pathways of *Aspergillus niger* under heat stress conditions.

Mutant	Trehalose content change at ambient temperature (mutant/wild type)	Heat stress temperature	Heat stress duration	Survival rate change (mutant/wild type)	References
ΔtpsA	44.0%	50 °C	60 min	74.5%	Wolschek and Kubicek (1997)
		55 °C	60 min	35.1%	
ΔtppB	32.2%	55 °C	60 min	44.9%	Svanström et al. (2014)
			120 min	5.3%	
ΔtreB	214.3%	50 °C	10 min	114.3%	Svanström and Melin (2013)
			20 min	140.0%	
			40 min	166.7%	

From the reverse perspective of “trehalose degradation,” this conclusion is further substantiated: mutants with a mutation in the *treB* gene, which encodes neutral trehalase, display impaired trehalose degradation, resulting in elevated intracellular trehalose levels. Consequently, their acquired thermotolerance during the initial stage of spore germination is 1.5 times greater than that of the wild type (Svanström and Melin, 2013) (Table 1). Collectively, these findings—derived from both the increase and decrease in trehalose content—demonstrate that trehalose is an essential protective molecule for *A. niger* in managing heat stress and maintaining cellular homeostasis.

Studies on *A. niger* have identified a distinct differential expression pattern of its trehalose synthesis genes in response to heat stress. Notably, the *tpsA* gene demonstrates constitutive expression, maintaining a basal level under normal growth conditions to provide essential heat protection for cells. In contrast, the *tpsB* gene functions as a typical heat-inducible gene (Wolschek and Kubicek, 1997). Upon exposure to heat shock, the transcriptional level of *tpsB* is rapidly and significantly upregulated, facilitating the substantial synthesis of trehalose to mitigate sudden environmental stress. This dual-gene regulatory mechanism—comprising a “housekeeping gene” that sustains basal levels and a “stress-responsive gene” that addresses stress—illustrates an effective strategy employed by *A. niger* to adapt to environmental fluctuations.

### 3.2 Mannitol metabolic pathway

Mannitol, the main compatible solute in *A. niger* conidia, accounts for 10–15% of their dry weight (Witteveen and Visser, 1995; Ruijter et al., 2003). In fungal cells, mannitol functions as a reserve carbon source, maintains intracellular osmotic balance (Chaturvedi et al., 1997), regulates the NAD/NADPH coenzyme balance (Dulermo et al., 2015; Gonçalves et al., 2019), and acts as an antioxidant to scavenge reactive oxygen species (ROS) induced by heat stress (Smirnoff and Cumbes, 1989).

The intracellular concentration of mannitol in *A. niger* is bidirectionally regulated by mannitol dehydrogenase (MTD) and mannitol 1-phosphate dehydrogenase (MPD): MTD, mainly encoded by the *mtdB* gene (Seekles et al., 2023), catalyzes the oxidation of mannitol to fructose (Aguilar-Osorio et al., 2010); MPD, encoded by the *mpdA* gene (Ruijter et al., 2003), reduces fructose-6-phosphate (F6P) to mannitol-1-phosphate (M1P) (Kiser and Niehaus, 1981), which is then dephosphorylated to form mannitol (Figure 2B).

The *mpdA* gene, encoding MPD, is critical for the mannitol synthesis pathway. Studies have clearly shown that the mannitol content in conidia of the *ΔmpdA* mutant is only 30% of that in the wild type (Ruijter et al., 2003), and its tolerance to heat stress is significantly reduced. Exogenous addition of mannitol can partially restore its stress-resistant phenotype, which effectively confirms that the synthesis and accumulation of mannitol are crucial for *A. niger* to resist heat damage.

Previous proteomic studies conducted by our team (Deng et al., 2020) demonstrated that under heat stress, the expression level of MPD (A0A100I4E3_ASPNG) in *A. niger* is significantly upregulated, whereas the expression level of MTD (A0A100IRN0_ASPNG) is significantly downregulated (Deng et al., 2020). This finding confirms that heat stress induces *A. niger* to activate a directional adaptive regulation of the mannitol metabolic pathway, leading to a reconstruction of metabolic balance characterized by enhanced mannitol synthesis efficiency and reduced degradation consumption. Consequently, this process drives intracellular mannitol accumulation. These results suggest that the mannitol metabolic pathway is a core functional pathway for *A. niger* in its response to heat stress.

## 4 Protein kinase A mediated metabolic regulation

In eukaryotes, cAMP serves as a second messenger by binding to the regulatory subunit of protein kinase A (PKA), which results in the release and activation of its catalytic subunit. The activated PKA catalytic subunit subsequently phosphorylates downstream target proteins. In *A. niger*, both PKA catalytic subunit (Bencina et al., 1997) and PKA regulatory subunit (Saudohar et al., 2002) have been cloned and characterized. This pathway acts as a central hub for sensing and responding to environmental changes, regulating a variety of processes, including cell growth, morphological differentiation, metabolism, and stress responses. Indeed, the cAMP/PKA pathway orchestrates numerous cellular functions in *Saccharomyces cerevisiae*, including adaptive responses such as thermotolerance (Reca et al., 2020).

Heat stress has been shown to significantly downregulate the mRNA expression levels of the *pkaC* gene in *A. niger*, which is accompanied by a notable decrease in the enzymatic activity of PKA (Benčina and Legiša, 2000). This regulatory mechanism may function as an adaptive strategy for *A. niger* under heat stress, aiming to prevent the hyperphosphorylation of intracellular proteins and thus improve its tolerance to elevated temperatures.

The PKA in *A. niger* regulates intracellular lipid metabolism by modulating triacylglycerol lipase (TGL) activity (Jernejc and Bencina, 2003). Impairment of PKA activity markedly reduces TGL activity, leading to the accumulation of neutral lipids as an energy reserve. Concurrently, phospholipid synthesis is inhibited due to the lack of PKA activity and precursor competition from neutral lipid accumulation. This results in decreased total phospholipid content, notably reducing levels of lysophosphatidylethanolamine (LPE), PE, and phosphatidylinositol (PI).

Furthermore, 6-phosphofructo-1-kinase (PFK), a downstream target of PKA (Legisa and Bencina, 1994), catalyzes the conversion of F6P to fructose-1,6-bisphosphate (FDP). This irreversible step is the sole rate-limiting reaction in glycolysis. Thus, PFK activity directly determines whether glucose carbon is funneled into energy production or diverted toward the synthesis of protective compounds like mannitol and trehalose (Figure 2A).

Collectively, these findings suggest that heat stress-induced downregulation of PKA activity in *A. niger* alters lipid metabolism, promoting neutral lipid accumulation while reducing phospholipid levels. Additionally, diminished PKA activity likely attenuates glycolysis via PFK, redirecting carbon flux toward the synthesis of compatible solutes such as mannitol and trehalose. This metabolic rewiring facilitates the accumulation of osmolytes, helping to maintain cellular osmotic balance and protein stability under heat stress.

## 5 Protein quality control

As key effectors in the heat stress response, the HSP family participates in protein quality control by exerting molecular chaperone functions, primarily encompassing the inhibition of abnormal protein aggregation, promotion of correct polypeptide folding, and mediation of misfolded protein degradation (Kim et al., 2013). Based on molecular weight, this family is classified into multiple subfamilies, including HSP100, HSP90, HSP70, HSP60, HSP40, and small-molecular-weight HSPs (Tutar and Tutar, 2010). Among these, the HSP70 and HSP104 subfamilies (members of the HSP100 family) play crucial roles in regulating fungal thermotolerance.

Genetic studies on *S. cerevisiae* have shown that both *hsp70* (Werner-Washburne et al., 1987) and *hsp104* (Sanchez and Lindquist, 1990; Sanchez et al., 1992) knockout strains exhibit significantly reduced thermotolerance compared to the wild-type strain, confirming the key roles of these two subfamilies in the heat stress response. Proteomic analyses (Deng et al., 2020) have demonstrated that the expression levels of HSP70 and HSP98/HSP104 in *A. niger* are significantly upregulated in response to heat stress. This finding closely parallels the results observed in *S. cerevisiae* (Jarnuczak et al., 2018). Such changes are likely among the key mechanisms facilitating adaptation to high-temperature environments.

Furthermore, the endoplasmic reticulum (ER) serves as the quality control hub for secretory protein folding. Both abnormal ER secretion and protein folding induced by heat shock trigger the unfolded protein response (UPR) (Miskei et al., 2009), which is initiated by the Ire1p-BiP system (BiP is also known as Kar2p, an Ire1p regulator belonging to the Hsp70p family) (Bertolotti et al., 2000; Kimata et al., 2007). This process enhances the ER’s protein-processing capacity by upregulating the expression of ER-localized chaperones (e.g., BiP) and foldases (e.g., protein disulfide isomerase, PDI).

Taking yeast as an example: under non-stress conditions, Ire1p binds tightly to the ER chaperone BiP; when a large number of unfolded proteins accumulate due to external stress, BiP dissociates from Ire1p to bind these unfolded proteins (Okamura et al., 2000). Subsequently, Ire1p oligomerizes and further activates itself (Credle et al., 2005). Activated Ire1p catalyzes the unconventional splicing of Hac1p mRNA (Fauzee et al., 2020), thereby promoting the synthesis of the transcription factor Hac1p. Finally, Hac1p is translocated to the nucleus and binds to the UPR response elements in the promoters of UPR target genes, which strongly induces the transcription of genes such as *bip* and *pdi* (Guerfal et al., 2010).

In *A. niger*, HacA—the homolog of Hac1p—has been identified, and it exhibits a similar splicing reaction to that of Hac1p (Mulder et al., 2004). Notably, the promoter region of the *bipA* gene in *A. niger* contains sequences homologous to heat shock elements (HSE) and UPR elements, and heat stress can directly induce its expression (Van Gemeren et al., 1997). HSE is a short DNA sequence located in the promoter region of HSP genes, serving primarily as a docking site for heat shock transcription factors (Hsf). Upon detection of heat stress, inactive Hsf undergoes trimerization and translocates into the nucleus. The activated Hsf trimers specifically recognize and bind to the HSE sequences upstream of HSP genes (Morano et al., 2012; Verghese Jacob et al., 2012). This binding resembles a key inserting into a lock and turning it; it activates RNA polymerase, thereby initiating the extensive transcription of downstream HSP genes into mRNA, which is subsequently translated into substantial quantities of HSP proteins (Xiao et al., 2022). A separate study further revealed that under ER stress, the induction of the *bipA* gene in *A. niger* does not depend on the transcription factor HacA (Davé et al., 2006). This phenomenon differs from the UPR mechanism in yeast, which may represent a protein quality control regulatory mechanism specific to *A. niger*.

## 6 Activation of the antioxidant defense system

Under normal physiological conditions, ROS can maintain cellular homeostasis and participate in regulating important biological processes such as cellular signal transduction (Sies and Jones, 2020). However, heat stress causes multifaceted damage to cells; one key physiological consequence is the induction of excessive intracellular ROS production, which triggers oxidative stress (Abrashev et al., 2008). This leads to damage to intracellular biomacromolecules including proteins, lipids, and nucleic acids, impairing cellular metabolism and normal physiological functions (Juan et al., 2021). To counteract this, fungi generally possess an antioxidant defense system composed of enzymatic and non-enzymatic components, which is capable of scavenging ROS and repairing ROS-induced damage. Among these components, the primary antioxidant enzymes are superoxide dismutase and catalase.

Under heat stress, *A. niger* can synergistically activate antioxidant defense mechanisms through multiple pathways to counteract oxidative damage and maintain the stability of cellular metabolism and physiological functions. On one hand, it upregulates the expression levels and enzymatic activities of superoxide dismutase and catalase (Bai et al., 2003; Abrashev et al., 2005, 2008, 2014; Deng et al., 2020); by directly scavenging excessive intracellular ROS and alleviating damage to biomacromolecules such as proteins and lipids, it copes with oxidative stress induced by heat stress. On the other hand, *A. niger* can respond to heat stimuli by activating the HSE and stress response element (STRE) in the promoter region of the alternative oxidase gene *aox1* (Honda et al., 2012), promoting the transcription of *aox1* and subsequent translation to generate alternative oxidase AOX1 (Hou et al., 2018; Deng et al., 2020). AOX1 can reduce the production of ROS in mitochondria by diverting mitochondrial electron flow (Maxwell et al., 1999), further minimizing the impact of oxidative damage on cells.

## 7 The potential role of the cell wall integrity pathway

The cell wall of *A. niger* primarily consists of a cross-linked polysaccharide network, with key components including chitin, β-1,3-glucan, and α-1,3-glucan (Kang et al., 2018). These fibrous polysaccharides establish the rigid core of the cell wall. Additionally, the cell wall incorporates several highly glycosylated proteins, such as mannoproteins, glycosylphosphatidylinositol (GPI)-modified cell wall proteins, and hydrophobins (De Groot et al., 2005). Under heat stress, the GPI-anchored cell wall organization protein Ecm33 is significantly upregulated in *A. niger* (Deng et al., 2020). This protein is essential for the proper ultrastructural organization of the fungal cell wall and the correct assembly of the outer mannoprotein layer. Deletion of the gene encoding Ecm33 activates the Cell Wall Integrity (CWI) pathway, and reduces thermotolerance (Gil-Bona et al., 2016).

Cell wall stress refers to a cellular physiological state induced by any factor that interferes with cell wall biosynthesis or compromises its structural integrity. To counteract various endogenous and exogenous stresses disrupting cell wall integrity, fungi have evolved an elaborate signal transduction system, namely the CWI signaling pathway. Although direct evidence that heat stress elicits cell wall stress and activates the CWI pathway in *A. niger* remains lacking, wavy septa and intrahyphal hyphae formation have been observed in *A. niger* under heat stress (Abrashev et al., 2014). Similarly, cell wall thickening has been reported in *Aspergillus fumigatus* (Fabri et al., 2021) and *Candida albicans* (Ikezaki et al., 2019) following heat stress. These ultrastructural alterations are generally recognized as hallmarks of cell wall stress.

A typical CWI pathway initiates with transmembrane sensor proteins (e.g., Wsc1-3, Mid2) localized on the plasma membrane, which sense physical deformation or damage to the cell wall (Jendretzki et al., 2011). The signal is subsequently transmitted to Rho family GTPases (e.g., Rho1), and activated Rho1 further activates protein kinase C (PKC). As an upstream initiator of the MAPK cascade, PKC sequentially phosphorylates and activates downstream MAPKKK, MAPKK, and ultimately the terminal MAPK (Slt2/Mpk1 in yeast and its homolog MpkA in *Aspergillus* species). Activated MpkA translocates into the nucleus, phosphorylates downstream transcription factors (e.g., Rlm1 in yeast and its homolog RlmA in *Aspergillus*), thereby initiating the expression of a suite of genes associated with cell wall synthesis, repair, and remodeling to ultimately accomplish compensatory reinforcement and repair of the cell wall (Levin, 2011; Samantaray et al., 2013).

While the classic CWI pathway model designates Wsc/Mid family proteins as sensors, the specific upstream sensor proteins in *A. niger* and their activation mechanisms under heat stress remain incompletely elucidated. However, studies on Rho GTPase family members localized between sensors and PKC have provided critical insights. Specifically, in addition to the core Rho1, RhoB and RhoD in *A. niger* are also involved in cell wall stress responses (Fiedler et al., 2014). This suggests that multiple upstream signals may converge on the CWI pathway, or these distinct Rho GTPases exert context-specific functions in different cellular regions or in response to diverse types of damage. Furthermore, the upregulation of cell division control protein 42 in proteomic datasets (Deng et al., 2020) indicates extensive activation and functional reorganization of the Rho GTPase family under heat stress.

Additionally, the transcription factors RlmA, MsnA, and CrzA are equally critical for *A. niger* to cope with cell wall stress (Fiedler et al., 2014). Among these, RlmA— the key transcription factor downstream of the CWI pathway—binds to specific sequences in the promoter regions of target genes upon activation, upregulating the expression of numerous genes involved in cell wall synthesis, repair, and remodeling (Damveld et al., 2005a), such as *agsA* (encoding α-1,3-glucan synthase) (Ram et al., 2004) and *gfaA* (encoding glutamine:fructose-6-phosphate amidotransferase) (Damveld et al., 2005b). As a “general stress” transcriptional factor, MsnA is extensively involved in various external stresses (Martínez-Pastor et al., 1996). Studies have shown that when PKA activity is inhibited, the Msn2/4 (yeast homologs of MsnA)-mediated stress response is enhanced, improving the thermotolerance of fungi (Li et al., 2018).

Notably, under heat stress, several enzymes with apparent cell wall-degrading activities are also upregulated, including β-1,6-glucanase, α,α-trehalose-glucan glucosidase TreA/Ath1, chitinases, and α-glucosidases (Deng et al., 2020). Similarly, *A. fumigatus* can rapidly accumulate HsfA in the early stage of heat stress, which inhibits the cell wall synthesis gene *ags2* (encoding α-1,3-glucan synthase) and simultaneously activates the expression of the cell wall remodeling gene *exg1* (encoding β-1,3-exoglucanase) (Fabri et al., 2021). This apparent paradox reflects the intrinsic nature of dynamic cell wall remodeling. Cell wall repair is not a simple process of structural component deposition; instead, it requires precisely regulated hydrolases to cleave old glycosidic bonds, creating space for the insertion and cross-linking of new polysaccharide chains to achieve cell wall remodeling and reinforcement. The upregulation of these hydrolases indicates that the cell wall is in a state of synchronized, highly active degradation and synthesis.

Similarly, *A. fumigatus* can rapidly accumulate HsfA in the early stage of heat stress, which inhibits the cell wall synthesis gene *ags2* (encoding α-1,3-glucan synthase) and simultaneously activates the expression of the cell wall remodeling gene *exg1* (encoding β-1,3-exoglucanase) (Fabri et al., 2021). This phenomenon seems contradictory but actually reflects the essence of dynamic cell wall remodeling. Cell wall repair is not a simple “adding materials” process; instead, it requires precisely regulated hydrolases to cleave old glycosidic bonds, creating space for the insertion and cross-linking of new polysaccharide chains, thereby achieving cell wall remodeling and reinforcement. The upregulation of these hydrolases indicates that the cell wall is in a highly active and controlled state where degradation and synthesis proceed simultaneously.

## 8 Discussion and prospects

**Figure 3:**
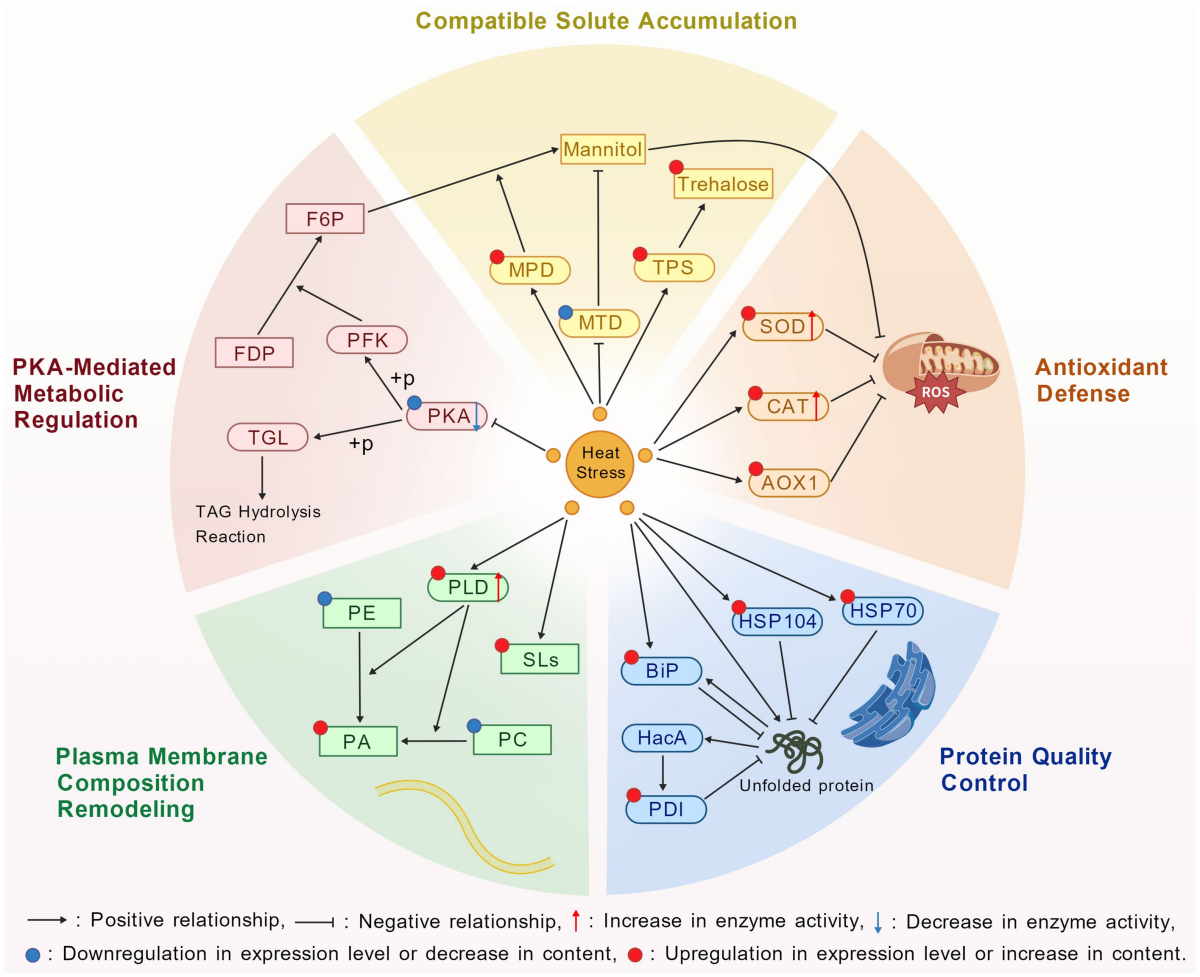
Mechanisms of heat stress response in *Aspergillus niger*.

This review systematically summarizes the response mechanisms of *A. niger* to heat stress, including membrane lipid rearrangement, PKA-mediated metabolic reprogramming, accumulation of compatible solutes, protein damage repair via HSPs and endoplasmic reticulum chaperones, activation of antioxidant enzyme systems to scavenge excessive ROS (Figure 3), as well as the potential role of the CWI pathway. Collectively, these processes act synergistically to enhance the resistance and adaptability of *A. niger* to heat stress. Despite substantial progress in current research on the heat stress response mechanisms of *A. niger*, numerous areas remain to be further explored.

In microbiological research, heat stress is typically classified into Short-Term Heat Shock (SHS) (or sudden heat shock) and Long-Term Heat Stress (LHS) (or prolonged exposure to elevated temperatures). SHS aims to simulate rapid and drastic temperature fluctuations in natural environments and investigate the immediate, transient physiological responses of cells. The temperature usually jumps sharply from the optimal growth temperature to a significantly higher level, even approaching or slightly exceeding the upper limit of the organism’s survival range. Such temperatures typically induce obvious protein denaturation. The time scale of SHS is generally on the minute level; the heat treatment duration employed in experiments can be as short as 20 s (Valentine et al., 2019), commonly 2 min (McClanahan and McEntee, 1986; Kanshin et al., 2015), or even longer (Liu et al., 2011). Research focus primarily lies on cellular heat sensing and heat signal transduction.

In contrast, LHS is designed to mimic the growth and adaptation processes of microorganisms in persistently high-temperature environments, such as within host organisms, industrial fermenters, or natural habitats under global warming. The temperature is usually set at a sustained level above the optimal growth temperature but is sublethal. This temperature significantly inhibits the growth rate yet still allows slow cell proliferation and adaptation. It aims to impose continuous selective pressure rather than cause immediate, large-scale cell death. The time scale of LHS typically ranges from hours to days (Gan et al., 2022). Research focuses more on the cellular mechanisms of heat acclimation, including stable alterations in gene expression patterns, metabolic network reprogramming, remodeling of cell membrane lipid composition, and global adjustments of the proteome. The ultimate goal is to establish a new physiological homeostasis capable of supporting cell growth under the new high-temperature norm.

Current research on *A. niger* has almost exclusively focused on long-term heat stress, lacking the capture of early key signaling events, which hinders the complete elucidation of the full pathway from heat sensing to response. For instance, regarding the cell membrane—one of the potential first structures to sense temperature changes—studies in *Saccharomyces cerevisiae* have demonstrated that sphingolipid intermediates (e.g., C₂₀-dihydrosphingosine, C₂₀-phytosphingosine, and ceramides) accumulate rapidly in the early stage of heat stress (Dickson et al., 1997; Jenkins et al., 1997). These sphingolipid molecules act as core signaling mediators of thermotolerance by activating the STRE and promoting the synthesis of the thermoprotectant trehalose (Dickson et al., 1997).

Additionally, abrupt temperature elevation triggers an outburst of ROS, causing extensive cellular damage. Therefore, the rapid and effective neutralization of ROS before the full activation of repair systems (e.g., HSPs) is a prerequisite for cell survival. Studies have confirmed that the intracellular pyruvate level in fungal cells increases rapidly and significantly within minutes after heat treatment, and this accumulation occurs prior to the activation of other ROS-scavenging mechanisms (e.g., changes in glutathione levels or upregulation of antioxidant enzyme gene expression) (Zhang et al., 2017). As an α-keto acid, pyruvate can directly scavenge H₂O₂ through non-enzymatic decarboxylation, with a reaction rate even faster than that of catalase (Zhang et al., 2018). Thus, further investigation is warranted to explore other mechanisms that can respond rapidly and exert protective effects under heat stress.

Furthermore, existing studies have not distinguished the differences in heat stress responses between mycelia and conidia. As dormant structures, conidia of *A. niger* inherently contain more compatible solutes as reserve carbon sources compared to vegetative hyphae (Ruijter et al., 2003). Meanwhile, the conidial cell wall is enriched in mannose and galactose but depleted in N-acetylglucosamine (van Munster et al., 2013), which also confers enhanced heat stress resistance. Beyond the carbohydrate backbone, the conidial cell wall also contains a melanin layer, which endows conidia with their characteristic color and increases resistance to environmental stresses (Latgé et al., 2005).

Based on the aforementioned issues, future research can be carried out around the following directions: First, at a finer time scale, techniques such as real-time quantitative PCR and dynamic protein tracking can be used to analyze the expression changes of key enzymes and membrane proteins involved in cell membrane lipid metabolism during the early stage of heat stress, thereby clarifying the initial signal transduction pathway of membrane heat sensing. Second, through multi-omics integration analysis (transcriptomics, lipidomics, metabolomics, etc.) combined with metabolic engineering validation, a global regulatory network of the heat stress response in *A. niger* can be constructed. Finally, dynamic studies can be performed on the same strain at different growth and developmental stages to correct the limitations of existing research conclusions and avoid misjudgment of mechanisms caused by single-stage studies.

In conclusion, research on the heat stress response of *A. niger* has advanced from phenotypic description to mechanistic exploration. In the future, through the integration of multiple disciplines to decipher the precise regulatory rules of this complex network, it will not only provide new insights into the biology of fungal environmental adaptability but also offer strong impetus for driving innovations in industrial biotechnology.
