# Supplementary material for: Research progress on heat stress response mechanisms in Aspergillus niger
Source: Front Microbiol. 2026 Feb 4;17:1750016. doi: 10.3389/fmicb.2026.1750016 (PMC12913586; doi:10.3389/fmicb.2026.1750016)
Supplement: Supplementary file 1 [file Table_1.docx]

Supplementary Material

Supplementary Table 1 Significantly differentially expressed plasma membrane component proteins under heat stress at 50℃. The protein name, accession number and alternate ID were from the Uniprot database. The alternate ID column represents the ID of the protein-codinggenes. The ratio takes values of ≥ 1.2 or ≤ 0.83, i.e., the Fold-change is ≥ 1.2 (including both up-regulation by ≥ 1.2-fold and down-regulation by ≥ 1.2-fold).

| Protein name | Accession numbers | Alternate ID | T-test p-value | Ratio(50℃/30℃) |
| --- | --- | --- | --- | --- |
| RAS small monomeric GTPase | A0A100IPK2_ASPNG | ABL_07431 | 0.0372342 | 3.665882628 |
| GPI-anchored cell wall organization protein Ecm33 | A0A117DX26_ASPNG | ABL_02084 | 0.000248254 | 3.546181978 |
| Transmembrane 9 superfamily member | A0A117E3A7_ASPNG | ABL_07847 | 0.00204131 | 2.188028129 |
| Phospholipase, PLC-D | A0A100IL38_ASPNG | ABL_05865 | 0.000309634 | 1.817626596 |
| Tyrosinase | A0A124BUX3_ASPNG | ABL_00606 | 0.00045084 | 1.801217976 |
| ABC bile acid transporter | A0A117DZ47_ASPNG | ABL_03647 | 2.21E-05 | 1.788207589 |
| Nucleoside transporter | A0A117DY97_ASPNG | ABL_02949 | 0.00317353 | 1.720890647 |
| Protein-S-isoprenylcysteine O-methyltransferase | A0A124BY10_ASPNG | ABL_06739 | 0.00172216 | 1.677601498 |
| Cell division control protein 42 | A0A100IRP8_ASPNG | ABL_08768 | 0.0121448 | 1.651793898 |
| Integral membrane protein | A0A100I2B3_ASPNG | ABL_00168 | 0.00201227 | 1.640885317 |
| Formate/nitrite transporter family protein | A0A117E3R2_ASPNG | ABL_08253 | 0.000170405 | 1.630680872 |
| Guanine nucleotide-binding protein subunit alpha | A0A100IIH6_ASPNG | ABL_04485 | 6.21E-05 | 1.59192677 |
| Purine nucleoside permease | A0A100IQJ3_ASPNG | ABL_08199 | 0.0171139 | 1.575643544 |
| Integral membrane protein | A0A100IRW0_ASPNG | ABL_08863 | 0.000814586 | 1.525275038 |
| Potassium ion channel Yvc1 | A0A124BVQ3_ASPNG | ABL_01856 | 0.000729306 | 1.522378772 |
| Choline transporter | A0A100I426_ASPNG | ABL_00641 | 0.00841555 | 1.52178333 |
| ABC multidrug transporter Mdr1 | A0A117E2B9_ASPNG | ABL_08039 | 0.000153593 | 1.505261926 |
| Phosphatidylinositol 4-kinase type II subunit alpha | A0A100I5B9_ASPNG | ABL_00995 | 0.000758899 | 1.497014096 |
| Stomatin family protein | A0A100IIT4_ASPNG | ABL_04170 | 0.00208119 | 1.494089617 |
| Calcium-transporting ATPase | A0A124BY56_ASPNG | ABL_07154 | 0.00132221 | 1.467622086 |
| Integral membrane protein | A0A117E3L3_ASPNG | ABL_09106 | 0.00243116 | 1.457023193 |
| Exocyst complex component Exo70 | A0A100I6B8_ASPNG | ABL_00857 | 0.00209794 | 1.452713065 |
| Lectin family integral membrane protein | A0A100IBV7_ASPNG | ABL_02621 | 0.00494034 | 1.428996692 |
| ATP-dependent bile acid permease | A0A100IEV9_ASPNG | ABL_03192 | 0.00420958 | 1.406846357 |
| Long-chain fatty acid transporter | A0A100ICR0_ASPNG | ABL_02824 | 0.000183209 | 1.393105425 |
| Phospholipase D | A0A100IFF6_ASPNG | ABL_03524 | 0.00146743 | 1.388418329 |
| Calcium channel subunit Cch1 | A0A100II07_ASPNG | ABL_04293 | 0.00165176 | 1.377156843 |
| Cyclopropane-fatty-acyl-phospholipid synthase | A0A100IT27_ASPNG | ABL_09480 | 0.00943179 | 1.37532069 |
| GTP-binding protein RHO3 | A0A100IPD4_ASPNG | ABL_07326 | 0.00549937 | 1.374728265 |
| Phosphatidylinositol phospholipase C | A0A100I4Y1_ASPNG | ABL_00458 | 0.00839278 | 1.372438083 |
| MFS phosphate transporter | A0A100IPB8_ASPNG | ABL_07556 | 0.00786111 | 1.342947819 |
| 14-alpha sterol demethylase Cyp51B | A0A117E3A2_ASPNG | ABL_09434 | 0.00257862 | 1.341102617 |
| ABC metal ion transporter | A0A100ING4_ASPNG | ABL_06772 | 0.000805748 | 1.339065798 |
| C2 domain protein | A0A100IS77_ASPNG | ABL_09040 | 0.000752746 | 1.318193773 |
| Membrane bound C2 domain protein vp115 | A0A100ISB5_ASPNG | ABL_09083 | 0.000475798 | 1.296090512 |
| Signal transduction protein Syg1 | A0A117DYJ9_ASPNG | ABL_03206 | 0.0218594 | 1.283218308 |
| SNARE domain protein | A0A117DYS1_ASPNG | ABL_02109 | 0.000524844 | 1.249701661 |
| ABC transporter | A0A117DW86_ASPNG | ABL_01487 | 0.0119343 | 1.249252742 |
| Scramblase family protein | A0A100IME3_ASPNG | ABL_06525 | 0.0087554 | 1.246517724 |
| Sulfate transporter family protein | A0A117DWV5_ASPNG | ABL_00501 | 0.0224823 | 1.242515116 |
| Phospholipase D1 | A0A117E342_ASPNG | ABL_07442 | 0.00101302 | 1.235413888 |
| Phosphatidylinositol 4-kinase | A0A117E3A1_ASPNG | ABL_09436 | 0.00819155 | 1.211743805 |
| MFS sugar transporter | A0A100IGS9_ASPNG | ABL_03910 | 0.00151347 | 0.81701092 |
| Amino acid transporter | A0A124BWT0_ASPNG | ABL_03490 | 0.00125177 | 0.804462162 |
| Phosphatidylinositol transporter | A0A117E025_ASPNG | ABL_03199 | 0.031455 | 0.801022488 |
| ABC transporter | A0A100ICU6_ASPNG | ABL_02825 | 0.0123398 | 0.800476867 |
| Integral membrane protein | A0A124BUS7_ASPNG | ABL_00391 | 0.0215022 | 0.782441374 |
| Calcium permease family membrane transporter | A0A100IKW6_ASPNG | ABL_05760 | 0.00461799 | 0.756571992 |
| SNARE domain protein | A0A100ISE3_ASPNG | ABL_09114 | 0.0062504 | 0.726071998 |
| MFS multidrug transporter | A0A100INX2_ASPNG | ABL_07086 | 0.000619712 | 0.722598379 |
| Patched sphingolipid transporter | A0A100IJE3_ASPNG | ABL_05005 | 0.0378714 | 0.689565789 |
| DUF1212 domain membrane protein | A0A117E1H0_ASPNG | ABL_06679 | 0.0403269 | 0.655620158 |
| Ammonium transporter | A0A100IRQ3_ASPNG | ABL_08785 | 0.0048331 | 0.639888512 |
| Integral membrane protein 25D9-6 | A0A100ICS4_ASPNG | ABL_02644 | 0.00250239 | 0.563396445 |
| Plasma membrane SNARE protein | A0A117DV90_ASPNG | ABL_00766 | 0.0023109 | 0.52652553 |
| G protein gamma subunit | A0A100I3V2_ASPNG | ABL_00256 | 0.000712418 | 0.47467841 |
| DUF803 domain membrane protein | A0A100I8A3_ASPNG | ABL_01356 | 0.00385638 | 0.468866894 |
